# Protein compound macrophage migration inhibitory factor for diagnosing postmenopausal osteoporosis

**DOI:** 10.3389/fchem.2026.1780639

**Published:** 2026-02-06

**Authors:** Chendi Wang, Fankai Huang, Hehuan Lai, Enzhao Cai, Danqing Ma, Yu Bai, Dengwei He, Zhenzhong Chen

**Affiliations:** 1 Department of Orthopedics, 5th Affiliated Hospital, Lishui Municipal Central Hospital, Wenzhou Medical College, Lishui, China; 2 Department of Orthopedics, Lishui Municipal Central Hospital, Lishui, China

**Keywords:** bone mineral density, bone turnover, diagnostic value, MIF, postmenopausal osteoporosis

## Abstract

Postmenopausal osteoporosis (PMOP) is a bone metabolic disorder characterized by reduced bone mass and deterioration of bone microarchitecture, and its early diagnosis is essential for improving clinical outcomes. However, reliable biochemical markers for the early detection of PMOP remain unavailable in current practice. Macrophage migration inhibitory factor (MIF) is an inflammation-related protein with oxidoreductase-like activity that participates in bone remodeling and metabolic regulation Mainly by activating the NF-κB signaling pathway. Nevertheless, its potential utility as a diagnostic compound in PMOP has not been fully clarified. The objective of this study was to investigate the plasma expression pattern of MIF in PMOP patients and explore its potential as an early diagnostic biomarker. Plasma MIF levels were significantly elevated in PMOP patients compared with individuals with normal bone mass (1.72 ng/mL vs. 0.58 ng/mL, p < 0.001). Moreover, MIF concentrations were negatively correlated with bone mineral density at the femoral neck (r = −0.548) and lumbar spine (r = −0.513), and positively correlated with bone turnover markers β-CTX (r = 0.417) and PINP (r = 0.350), suggesting that elevated MIF may reflect enhanced bone resorption and disruption of bone metabolic homeostasis. Further multivariate analyses identified MIF as an independent risk factor for PMOP and demonstrated its substantial diagnostic value. In conclusion, MIF,an inflammation-associated protein with oxidoreductase-like catalytic properties,may serve as a biochemical indicator of abnormal bone metabolism, providing a potential molecular basis for the early diagnosis of PMOP.

## 1 Introduction

Osteoporosis (OP) is a systemic metabolic bone disease characterized by reduced bone mass, deterioration of bone microarchitecture, and decreased bone strength, ultimately resulting in increased bone fragility and an elevated risk of fractures (Compston et al., 2019). Estrogen plays a critical role in maintaining bone metabolic homeostasis by inhibiting osteoclast activity and promoting the proliferation and differentiation of osteoblasts, thereby preserving the balance of bone remodeling (Eastell et al., 2016). In postmenopausal women, a sharp decline in estrogen levels leads to markedly increased bone resorption and rapid bone loss, rendering this population consistently at high risk for osteoporosis (Richelson et al., 1984; Sözen et al., 2017). The prevalence of postmenopausal osteoporosis (PMOP) and the incidence of osteoporotic fractures are significantly higher in women than in men (Morin et al., 2025). Approximately one-third of elderly women will suffer an osteoporotic fracture, and the risk continues to increase with advancing age (Hernlund et al., 2013). With the rapidly aging global population, PMOP has become a major medical and economic burden and is now a pressing global public health issue (Shen et al., 2022).

At present, the evaluation of bone mineral density (BMD) is still considered the “gold standard” for diagnosing osteoporosis (Mederle et al., 2018). Commonly used measurement techniques include quantitative computed tomography (QCT), dual-energy X-ray absorptiometry (DXA) and quantitative ultrasound (QUS) (Blake and Fogelman, 2007; Glüer, 1997; Link, 2012). However, BMD primarily reflects changes in bone mass and lacks sensitivity in detecting early bone microarchitectural deterioration associated with initial bone loss (Rizzoli, 2010). Moreover, BMD measurements are susceptible to interference from factors such as osteophyte formation and vascular or soft tissue calcification (Bristow et al., 2019). In recent years, as research on bone turnover markers (BTMs) has advanced, an increasing body of evidence has shown that BTMs have the ability to indicate the state of bone remodeling before a notable decrease in bone mineral density occurs. Owing to their advantages of being noninvasive, dynamic, and highly reproducible, BTMs have been gradually incorporated into clinical practice (Lewiecki, 2010; Vasikaran et al., 2011). Commonly used BTMs include procollagen type I N-terminal propeptide (PINP) and the β-C-terminal telopeptide of type I collagen (β-CTX) (Naylor and Eastell, 2012). However, due to limitations related to specificity, sensitivity, biological stability, and inter-individual variability among different BTMs, they are currently insufficient to serve as standalone diagnostic tools for osteoporosis (Lorentzon et al., 2019). Therefore, it is imperative to identify predictive markers with enhanced diagnostic accuracy that can effectively indicate abnormal bone metabolism in its initial stages.

**FIGURE 1:**
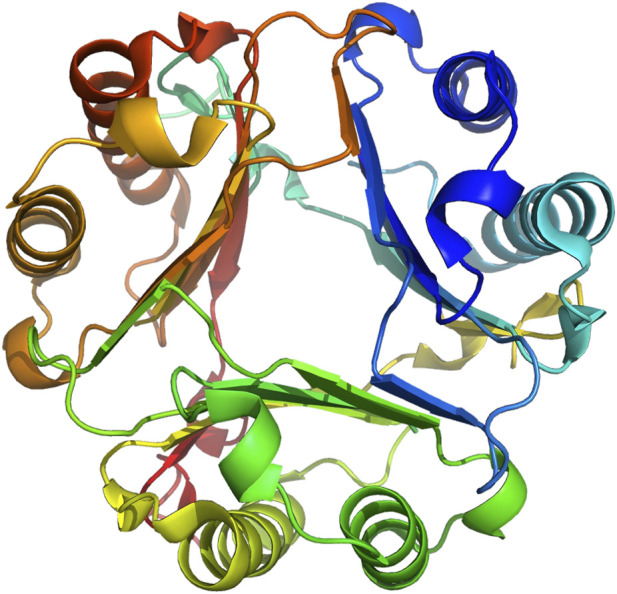
Structure of human macrophage migration inhibitory factor (MIF).

Macrophage migration inhibitory factor (MIF) is a secreted protein composed of 115 amino acids with a molecular weight of 12.5 kDa and is classified as a highly conserved, non-glycosylated protein (Bernhagen et al., 1994) (Figure 1). As one of the earliest identified inflammation-related mediators, MIF exhibits a high degree of sequence and functional conservation across multiple species. It exerts its biological activities through autocrine or paracrine mechanisms and plays a central regulatory role in immune modulation and the maintenance of tissue homeostasis (Calandra and Roger, 2003). MIF enhances the host immune defense by activating macrophages to promote the production of pro-inflammatory cytokines, including TNF-α and IL-2, and by facilitating pathogen clearance through the induction of nitric oxide (NO), cyclooxygenase-2 (COX-2), and highly efficient antigen presentation (Calandra and Roger, 2003; Lue et al., 2002; Youness et al., 2025). In addition, MIF exerts its biological functions through binding to receptors such as CD74, CD44, and CXCR2, thereby activating multiple intracellular signaling pathways, including nuclear factor-κB (NF-κB) and mitogen-activated protein kinases (MAPKs) (Aliyarbayova et al., 2025). Through these mechanisms, MIF plays a comprehensive regulatory role in cell growth, differentiation, survival, and inflammatory responses, and consequently participates in various physiological processes, such as tissue repair, neuroprotection, maintenance of cardiac function, and metabolic regulation (Camacho Meza et al., 2025; Sumaiya et al., 2022). MIF has been demonstrated to act as a crucial regulator in the initiation and progression of numerous inflammation-associated and metabolic disorders. Accumulating evidence indicates that MIF is of significant pathological relevance and potential diagnostic and therapeutic value in the occurrence, progression, and prognostic evaluation of diseases such as rheumatoid arthritis, asthma, diabetes mellitus, atherosclerosis, and various malignancies (Schindler et al., 2018).

In the past few years, there has been a growing focus on the significant impact of chronic inflammation in the development and advancement of osteoporosis. A mounting volume of evidence suggests that inflammatory responses can profoundly influence bone remodeling through osteoimmunological mechanisms, thereby promoting the development and progression of osteoporosis (Arron and Choi, 2000; Takayanagi, 2007; Takayanagi, 2009). Among various inflammation-related mediators, MIF, as a multifunctional proinflammatory cytokine, is acknowledged to play a critical part in modulating bone metabolic processes. Although some studies have indicated that MIF may exert MIF may have osteogenic effects in certain circumstances (Li et al., 2018; Kobayashi et al., 2011; Li et al., 2025), the prevailing evidence suggests that, within inflammation-related microenvironments, MIF primarily stimulates bone resorption while impeding bone formation. Studies on the mechanisms have shown that MIF boosts the growth and specialization of osteoclasts and intensifies their bone-resorbing activity by activating the nuclear factor-B (NF-B) signaling pathway, while also inhibiting osteoblast differentiation and bone formation simultaneously (Gu et al., 2015; He et al., 2023; Shi et al., 2023). Sustained or excessive activation of the NF-κB pathway disrupts the dynamic balance between bone resorption and bone formation, leading to bone loss and deterioration of bone microarchitecture, ultimately resulting in osteoporosis (Abu-Amer, 2013; Otero et al., 2012). Animal studies further support these findings, showing that MIF delays fracture healing by enhancing osteoclast activity (Kobayashi et al., 2011), and that transgenic mice with MIF overexpression exhibit more pronounced bone loss (Onodera et al., 2006). Building upon these findings, our previous work demonstrated that the MIF inhibitors Chicago sky blue 6B (CSB6B) and 4-iodo-6-phenylpyrimidine (4-IPP) effectively suppress the pharmacological activity of MIF and selectively inhibit the NF-κB signaling pathway, thereby markedly attenuating osteoclastogenesis and promoting osteoblast differentiation (Jin et al., 2021; Zheng et al., 2019). These results highlight a pivotal regulatory role of MIF in bone metabolic imbalance. Collectively, the above evidence provides experimental support for MIF as a promising therapeutic focus for osteolytic bone diseases, and also indicates that MIF may be intricately involved in the development of osteoporosis, with potential significance as a predictive biochemical biomarker.

Although previous findings indicating a strong correlation between elevated MIF levels, reduced bone mineral density and increased bone turnover in postmenopausal women (Kim et al., 2016), its clinical diagnostic value in PMOP has not yet been systematically evaluated. At present, it remains unclear whether MIF can serve as an independent predictive indicator reflecting abnormal bone metabolism, as well as its diagnostic performance and optimal cutoff value for discriminating between normal bone mass and osteoporotic status. These uncertainties have limited its further application in clinical practice. Therefore, the objective of this study was to quantify plasma MIF levels in women with postmenopausal status and, in combination with assessment of BMD and bone turnover biochemical markers, to systematically analyze the relationship between MIF and PMOP and to evaluate its independent predictive value after adjusting for multiple variables. Furthermore, our goal was to evaluate the potential of MIF as a biochemical indicator for early detection of PMOP, providing additional theoretical backing for risk evaluation and focused intervention in PMOP.

## 2 Materials and methods

### 2.1 Study design and participants

**FIGURE 2:**
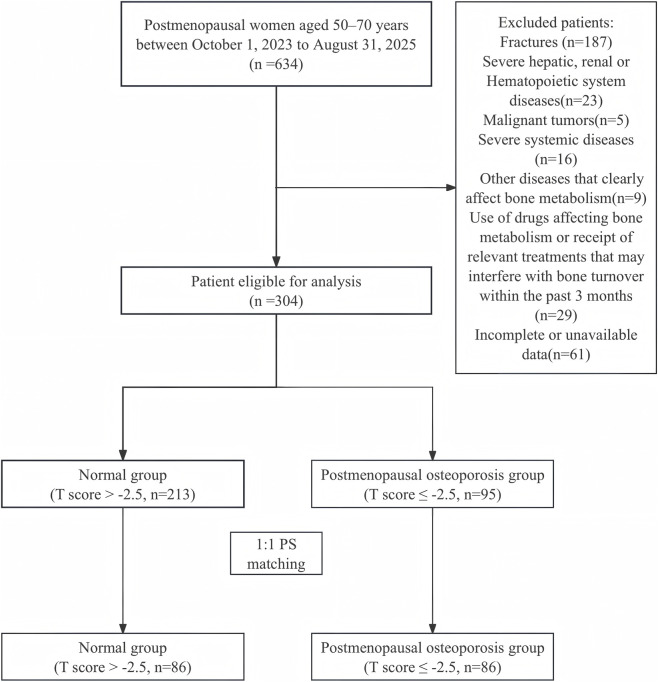
Diagram showing the process of patient selection. Note: PS matching: propensity score matching,PSM.

This study was designed as a cross-sectional observational study aimed at comparing plasma MIF levels and related bone metabolism indicators among postmenopausal women with different bone mineral density statuses, and to evaluate their associations with postmenopausal osteoporosis (PMOP) as well as their diagnostic value. Participants were consecutively recruited from postmenopausal women who attended Lishui Central Hospital between October 2023 and August 2025 (Figure 2). PMOP was diagnosed by adhering to the osteoporosis-related diagnostic protocols released by the World Health Organization (WHO) (Kanis, 1994). In this study, menopause was defined as the permanent cessation of menstruation resulting from the loss of ovarian follicular function, confirmed by the absence of menstruation for at least 12 consecutive months, after excluding menstrual abnormalities caused by pregnancy, endocrine disorders, medications, or other pathological or physiological factors (Sarri et al., 2015). The eligibility criteria encompassed women aged 50–70 years, with a menopausal duration exceeding 3 years, and normal glucose metabolism. Exclusion criteria comprised a history of osteoporotic or traumatic fractures, severe hepatic, renal, or hematological disorders, malignancies, serious systemic diseases, or other conditions impacting bone metabolism, as well as the use of medications or treatments that could affect bone metabolism or turnover within the previous 3 months, and incomplete or missing clinical data. This study was approved by the Ethics Committee of Lishui Central Hospital, Zhejiang Province (approval number: 2020-17), and was conducted in accordance with the Declaration of Helsinki. Written informed consent was obtained from all participants prior to enrollment.

### 2.2 Baseline data collection

All participants underwent standardized baseline data collection at enrollment. Documented general demographic data and clinical characteristics encompassed age and body mass index (BMI), years since menopause (YSM), medical history, and lifestyle-related factors. Age was recorded as the actual age (in years) at the time of enrollment. We determined BMI by measuring height and weight and using the formula: weight (kg) divided by height squared (m). We defined years since menopause (YSM) as the time period between the age at menopause and the age at enrollment. A history of hypertension was documented based on a confirmed previous diagnosis or current use of antihypertensive medications. Smoking and alcohol consumption histories were obtained through a structured questionnaire. We defined smoking history as current or previous smoking behavior, and alcohol consumption history as current or previous regular alcohol intake. When available, we verified the collected information against participants’ medical records to ensure accuracy.

### 2.3 Bone mineral density measurement

We measured bone mineral density (BMD) using dual-energy X-ray absorptiometry (DXA) at the lumbar spine (L1–L4) and femoral neck. DXA assessments were performed using a Prodigy Pro densitometer (GE Medical Systems, Monterrey, Mexico). We calculated T-scores based on the reference database provided by the manufacturer and expressed BMD values in g/cm^2^. According to the WHO diagnostic criteria for osteoporosis, participants were classified into two groups based on the lowest T-score obtained at any measured site: the non-osteoporosis group (Normal group, T-score > −2.5) and the postmenopausal osteoporosis group (PMOP group, T-score ≤ −2.5).

### 2.4 Measurement of bone turnover markers

Fasting venous blood samples were collected from all participants between 8:00 and 10:00 a.m. Serum was obtained by centrifugation and used for subsequent analyses. According to the manufacturers’ instructions, We measured serum levels of β-C-terminal telopeptide of type I collagen (β-CTX), procollagen type I N-terminal propeptide (PINP), parathyroid hormone (PTH), and osteocalcin (OC) using an electrochemiluminescence immunoassay (ECLIA) on a Cobas e602 automated analyzer (Roche Diagnostics, Shanghai, China). We measured serum 25-hydroxyvitamin D3 [25 -OH-D3] levels using a chemiluminescent microparticle immunoassay (CMIA) on an Alinity i automated analyzer (Abbott Diagnostics, Ireland). All laboratory measurements were performed by trained personnel in the hospital’s clinical laboratory. The testing procedures strictly followed the manufacturers’ protocols, and comprehensive quality control was implemented throughout the analytical process.

### 2.5 Data analysis

The crystal structure of MIF (PDB ID: 1GD0) was downloaded from the Protein Data Bank (https://www.rcsb.org/versions/1GD0).PyMOL (v2.5; Schrödinger, LLC) was employed to visualize the homotrimeric conformation of MIF. All statistical analyses were conducted using SPSS Statistics 26.0 (IBM, Armonk, NY, USA) in conjunction with GraphPad Prism 10.0 (GraphPad Software,San Diego, CA, USA),The distributional properties of continuous variables were examined using the Shapiro–Wilk test. Variables that followed a normal distribution are reported as mean ± standard deviation (SD),and differences between groups were assessed using the independent-samples t-test. For variables that did not meet normality assumptions, data are summarized as median with interquartile range [M (Q1,Q3)],and intergroup comparisons were performed using the Mann–Whitney U test. Categorical data are presented as counts and proportions, with group differences evaluated using either the chi-square test or Fisher’s exact test, as appropriate. To reduce the influence of potential confounding variables, propensity score matching (PSM) was applied with a 1:1 ratio based on age, BMI, and years since menopause. The caliper was set at 0.2, and matching was done without replacement. Further statistical analyses were performed after matching. Pearson or Spearman correlation analyses were used to examine the relationships between plasma MIF levels and bone mineral density (BMD) as well as bone turnover markers. Multivariate logistic regression was utilized to determine the independent associations between MIF concentrations, relevant clinical factors, and the risk of postmenopausal osteoporosis (PMOP). Receiver operating characteristic (ROC) curve analysis assessed the diagnostic accuracy of plasma MIF levels for PMOP.

## 3 Results

### 3.1 Study population characteristics and propensity score matching (PSM)

A total of 308 postmenopausal women were included in this study. Based on bone mineral density (BMD) measurements obtained by DXA, participants were divided into two groups: the PMOP group (n = 95) and the normal bone mass (Normal) group (n = 213). Baseline analysis revealed statistically significant differences between the two groups in age, BMI, years since menopause, lumbar spine and femoral neck BMD, and levels of PTH, 25 -OH-D_3_,β-CTX, and PINP (p < 0.05). Nevertheless, there were no notable disparities noted between the two cohorts in relation to the presence of hypertension, smoking, alcohol intake, and levels of osteocalcin (OC) (p > 0.05) (Table 1).

**TABLE 1:** Clinical baseline characteristics of the study subjects.

Parameters	Total (n = 308)	Normal (n = 213)	PMOP (n = 95)	Statics	*P*	SMD
Age (years),Mean ± SD	59.26 ± 4.92	58.69 ± 4.83	60.56 ± 4.91	t = −3.126	0.002	0.381
BMI(kg/m2),Mean ± SD	24.17 ± 3.50	24.97 ± 3.52	22.36 ± 2.70	t = 6.437	0.001	−0.966
Years since menopause, M(Q_1_,Q_3_)	10.00 (6.00,13.00)	8.00 (5.00,12.00)	11.00 (9.00,14.00)	Z = −4.335	0.001	0.570
Hypertension,n (%)	​	​	​	χ^2^ = 1.365	0.243	​
No	190 (61.69)	136 (63.85)	54 (56.84)	​	​	−0.141
Yes	118 (38.31)	77 (36.15)	41 (43.16)	​	​	0.141
Smoking history,n (%)	​	​	​	χ^2^ = 0.000	1.000	​
No	302 (98.05)	209 (98.12)	93 (97.89)	​	​	−0.016
Yes	6 (1.95)	4 (1.88)	2 (2.11)	​	​	0.016
Drinking history,n (%)	​	​	​	χ^2^ = 0.146	0.703	​
No	269 (87.34)	185 (86.85)	84 (88.42)	​	​	0.049
Yes	39 (12.66)	28 (13.15)	11 (11.58)	​	​	−0.049
Lumbar BMD (g/cm2),Mean ± SD	0.90 ± 0.23	1.00 ± 0.20	0.69 ± 0.09	t = 18.511	0.001	−3.421
Femoral neck BMD (g/cm2),Mean ± SD	0.90 ± 0.15	0.94 ± 0.15	0.79 ± 0.10	t = 10.660	0.001	−1.569
PTH (pg/mL),M(Q_1_,Q_3_)	42.10 (33.30,53.80)	41.10 (31.50,51.20)	45.10 (35.45,61.45)	Z = −3.043	0.002	0.383
25 -OH-D_3_ (ng/mL), M(Q_1_,Q_3_)	19.20 (15.20,23.77)	20.30 (15.40,24.60)	17.80 (14.95,21.95)	Z = −2.814	0.005	−0.431
OC (ng/mL),M(Q_1_,Q_3_)	17.91 (14.06,22.64)	17.55 (13.96,22.47)	18.23 (14.28,23.55)	Z = −0.571	0.568	0.088
Β-CTX (pg/mL), M(Q_1_,Q_3_)	491.50 (352.52,738.25)	417.00 (320.00,584.00)	783.00 (525.50,849.50)	Z = −8.097	0.001	1.049
PINP(ng/mL),M(Q_1_,Q_3_)	58.64 (43.31,75.39)	52.81 (41.81,67.31)	74.48 (64.34,82.25)	Z = −6.561	0.001	0.879
MIF(ng/mL),M(Q_1_,Q_3_)	0.81 (0.49,1.52)	0.59 (0.43,0.99)	1.72 (1.42,1.99)	Z = −10.649	0.001	1.393

t: t-test, Z: Mann-Whitney test, χ2: Chi-square test.

SD: standard deviation, M: median, Q_1_: first Quartile, Q_3_: 3rd Quartile.

p-value was significant when less than 0.05.

BMI, body mass index; BMD, bone mineral density; PTH, parathyroid hormone; 25-OH-D_3_, 25-hy-droxyvitamin D_3_; OC, osteocalcin; β-CTX, β-C-terminal telopeptide of type 1 collagen; PINP, procollagen type I N-propeptide; MIF, macrophage migration inhibitory factor.

Given that participants in the PMOP group were characterized by older age, longer years since menopause, and lower BMI, these baseline imbalances were considered potential confounding factors that might bias the intergroup comparisons. In order to reduce potential confounding bias, a 1:1 PSM method was utilized, taking into account age, BMI, and YSM. Following the matching process, a total of 172 participants were selected, with 86 in each of the Normal and PMOP groups (Table 2). The matching results showed no significant differences between the two groups in age (61.24 ± 4.65 years vs. 60.56 ± 5.06 years, p = 0.356), BMI (22.98 ± 2.79 kg/m^2^ vs. 22.64 ± 2.69 kg/m^2^, p = 0.416), years since menopause [median (Q_1_, Q_3_): 11.00 (7.25, 16.00) years vs. 11.00 (8.25, 14.00) years, p = 0.723], as well as in the history of hypertension, smoking, and alcohol consumption (p > 0.05). The results suggest that the baseline characteristics of the two groups were effectively matched.

**TABLE 2:** Clinical baseline characteristics after propensity score matching.

Parameters	Total (n = 172)	Normal (n = 86)	PMOP (n = 86)	Statics	*P*	SMD
Age (years),Mean ± SD	60.90 ± 4.85	61.24 ± 4.65	60.56 ± 5.06	t = 0.926	0.356	−0.136
BMI(kg/m2),Mean ± SD	22.81 ± 2.74	22.98 ± 2.79	22.64 ± 2.69	t = 0.815	0.416	−0.127
Years since menopause, M(Q_1_,Q_3_)	11.00 (8.00,14.00)	11.00 (7.25,16.00)	11.00 (8.25,14.00)	Z = −0.355	0.723	−0.089
Hypertension,n (%)	​	​	​	χ^2^ = 1.175	0.278	​
No	101 (58.72)	54 (62.79)	47 (54.65)	​	​	−0.163
Yes	71 (41.28)	32 (37.21)	39 (45.35)	​	​	0.163
Smoking history,n (%)	​	​	​	χ^2^ = 0.000	1.000	​
No	168 (97.67)	84 (97.67)	84 (97.67)	​	​	0.000
Yes	4 (2.33)	2 (2.33)	2 (2.33)	​	​	0.000
Drinking history,n (%)	​	​	​	χ^2^ = 0.488	0.485	​
No	151 (87.79)	74 (86.05)	77 (89.53)	​	​	0.114
Yes	21 (12.21)	12 (13.95)	9 (10.47)	​	​	−0.114
Lumbar BMD (g/cm2),Mean ± SD	0.76 ± 0.12	0.83 ± 0.10	0.68 ± 0.09	t = 10.641	0.001	−1.800
Femoral neck BMD (g/cm2),Mean ± SD	0.91 ± 0.17	1.03 ± 0.16	0.80 ± 0.09	t = 11.502	0.001	−2.436
PTH (pg/mL),M(Q_1_,Q_3_)	42.10 (32.72,53.80)	40.90 (29.52,52.05)	43.35 (35.23,59.65)	Z = −2.148	0.032	0.342
25- (OH)-D_3_ (ng/mL), M(Q_1_,Q_3_)	18.80 (15.67,23.77)	20.95 (16.10,25.18)	18.10 (15.62,22.58)	Z = −2.424	0.015	−0.575
OC (ng/mL),M(Q_1_,Q_3_)	18.19 (14.38,23.23)	18.15 (14.49,23.57)	18.19 (14.09,22.17)	Z = −0.421	0.674	−0.012
β-CTX (pg/mL),M(Q_1_,Q_3_)	533.50 (383.25,814.75)	416.00 (339.00,502.75)	800.00 (592.50,874.50)	Z = −7.851	0.001	1.311
PINP(ng/mL),M(Q_1_,Q_3_)	64.78 (48.89,77.50)	53.14 (42.55,66.52)	74.55 (64.36,82.00)	Z = −6.025	0.001	0.997
MIF(ng/mL),M(Q_1_,Q_3_)	1.14 (0.56,1.84)	0.58 (0.44,1.03)	1.72 (1.40,1.97)	Z = −8.353	0.001	1.337

t: t-test, Z: Mann-Whitney test, χ2: Chi-square test.

SD: standard deviation, M: median, Q_1_: first Quartile, Q_3_: 3rd Quartile.

p-value was significant when less than 0.05.

BMI, body mass index; BMD, bone mineral density; PTH, parathyroid hormone; 25-OH-D_3_, 25-hy-droxyvitamin D_3_; OC, osteocalcin; β-CTX, β-C-terminal telopeptide of type 1 collagen; PINP, procollagen type I N-propeptide; MIF, macrophage migration inhibitory factor.

### 3.2 Comparison of plasma MIF levels

**FIGURE 3:**
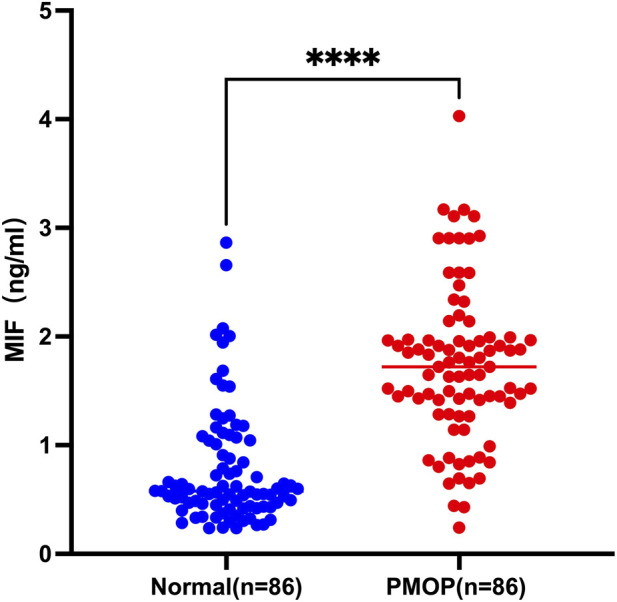
Comparison of plasma MIF concentrations. Plasma MIF concentrations were measured using ELISA kits. Data showed skewed distribution and were expressed as median (minimum, maximum), comparisons between the two groups were performed using the Mann-Whitney test. **** indicates p < 0.0001.

After performing propensity score matching, we assessed plasma MIF levels using ELISA in both groups. The results showed that the median plasma MIF concentration [Q_1_, Q_3_] in the PMOP group was 1.72 (1.40, 1.97) ng/mL, significantly exceeding that of the Normal group [0.58 (0.44, 1.03) ng/mL]. The difference was statistically significant (Z = −8.353, p < 0.001) (Table 2; Figure 3). The results indicate a substantial elevation in plasma MIF levels among individuals diagnosed with postmenopausal osteoporosis, highlighting its potential role in the condition.

### 3.3 Correlation analysis between plasma MIF levels and bone mineral density and bone metabolism markers

**FIGURE 4:**
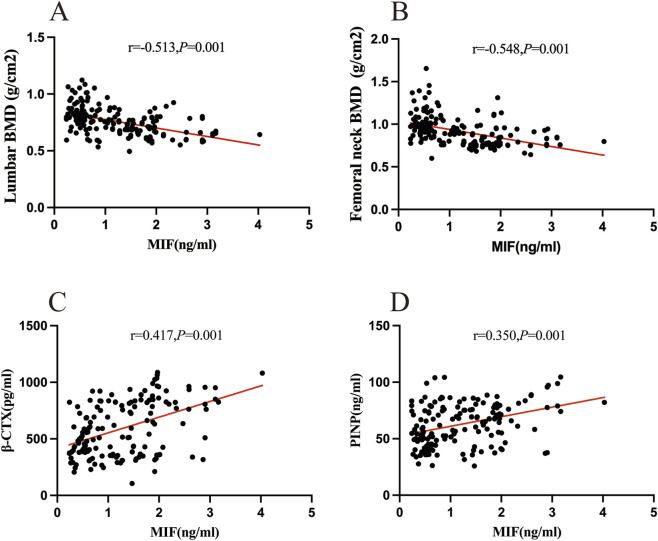
Correlations of plasma MIF concentrations with BMD values and bone turnover markers in PMOP patients. Data normality was tested by the Shapiro–Wilk method. Lumbar spine BMD, femoral neck BMD followed normal distribution; MIF, β-CTX and PINP were non-normally distributed, and their correlations were analyzed using r-Spearman correlation coefficient. r-Spearman correlation coefficient analyses of the relationships between plasma MIF concentrations and lumbar spine BMD **(A)**, femoral neck BMD **(B)**, β-CTX **(C)**,PINP **(D)** in PMOP patients.

Spearman correlation analysis were performed to investigate the potential associations between plasma macrophage migration inhibitory factor (MIF) concentrations and skeletal parameters (Table 3). The results showed that higher plasma MIF levels were significantly correlated with lower bone mineral density at both the femoral neck (r = 0.548, p < 0.001) and the lumbar spine (L1-L4) (r = 0.513, p < 0.001), indicating a clear inverse relationship between circulating MIF and regional BMD. Conversely, plasma MIF concentrations were positively correlated with markers of bone turnover, including -CTX (r = 0.417, p < 0.001) and PINP (r = 0.350, p < 0.001), suggesting a connection between elevated MIF levels and increased bone remodeling activity. However, there were no statistically significant correlations between plasma MIF levels and parathyroid hormone (PTH) or osteocalcin (OC) (p > 0.05) (Table 4; Figure 4).

**TABLE 3:** Correlation of Plasma MIF with BMD and bone turnover markers in PMOP patients after propensity score matching.

Parameters	r-Spearman	*P*
Lumbar BMD (g/cm2)	−0.513	0.001
Femoral neck BMD (g/cm2)	−0.548	0.001
β-CTX (pg/mL)	0.417	0.001
PINP(ng/mL)	0.350	0.001
PTH (pg/mL)	0.034	0.656
25 -OH-D_3_ (ng/mL)	−0.090	0.239
OC (ng/mL)	0.134	0.080

p-value was significant when less than 0.05.

BMD, bone mineral density; β-CTX, β-C-terminal telopeptide of type 1 collagen; PINP, procollagen type I N-propeptide; PTH, parathyroid hormone; 25-OH-D_3_, 25-hy-droxyvitamin D_3_; OC, Osteocalcin.

**TABLE 4:** Multivariate logistic regression analysis of independent influencing factors for PMOP after propensity score matching.

Variable	β	S.E	Z	*P*	OR (95%CI)
Constant term	17.722	6.735	2.632	**0.009**	​
PTH (pg/mL)	−0.002	0.02	−0.086	0.932	0.998 (0.960∼1.038)
25 -OH-D_3_ (ng/mL)	−0.098	0.07	−1.403	0.161	0.906 (0.790∼1.040)
β-CTX (pg/mL)	0.005	0.002	2.334	**0.020**	1.005 (1.001∼1.009)
PINP (ng/mL)	0.063	0.024	2.596	**0.009**	1.065 (1.015∼1.116)
MIF (ng/mL)	1.690	0.544	3.108	**0.002**	5.421 (1.867∼15.741)
Lumbar spine BMD × 10 (g/cm2)	−1.458	0.408	−3.573	**0.001**	0.233 (0.105∼0.518)
Femoral neck BMD × 10 (g/cm2)	−1.523	0.55	−2.767	**0.006**	0.218 (0.074∼0.641)

Hosmer-Lemeshow = 13.485, P = 0.096 > 0.05, model fit acceptable.

p-value was significant when less than 0.05.

PTH, parathyroid hormone; 25-OH-D_3_, 25-hy-droxyvitamin D_3_; β-CTX, β-C-terminal telopeptide of type 1 collagen; PINP, procollagen type I N-propeptide; BMD, bone mineral density; MIF, macrophage migration inhibitory factor.

All analyses were conducted after propensity score matching.

Bold values indicate statistically significant differences (p < 0.05).

### 3.4 Multivariate logistic regression analysis of risk factors for PMOP

All regression analyses were performed in the propensity score–matched cohort. Multivariate logistic regression analysis was performed with PMOP status as the dependent variable (PMOP = 1, non-PMOP = 0). PTH, 25 -OH-D_3_,β-CTX, PINP, plasma MIF levels, lumbar spine BMD, and femoral neck BMD were included as independent variables in the model (Table 4). The adequacy of the model fit was demonstrated by the Hosmer–Lemeshow goodness-of-fit test (χ^2^ = 13.485, p = 0.096). The regression analysis demonstrated that plasma MIF levels independently increase the risk of PMOP (β = 1.690, Z = 3.108, p = 0.002), with an odds ratio (OR) of 5.421 (95% confidence interval [CI]: 1.867–15.741). In addition, β-CTX (OR = 1.005, 95% CI: 1.001–1.009, p = 0.020) and PINP (OR = 1.065, 95% CI: 1.015–1.116, p = 0.009) were also identified as independent risk factors for PMOP. Conversely, lumbar spine BMD (OR = 0.233, 95% CI: 0.105–0.518, p < 0.001) and femoral neck BMD (OR = 0.218, 95% CI: 0.074–0.641, p = 0.006) were determined to be protective factors against PMOP.

### 3.5 Diagnostic performance of plasma MIF for PMOP

**FIGURE 5:**
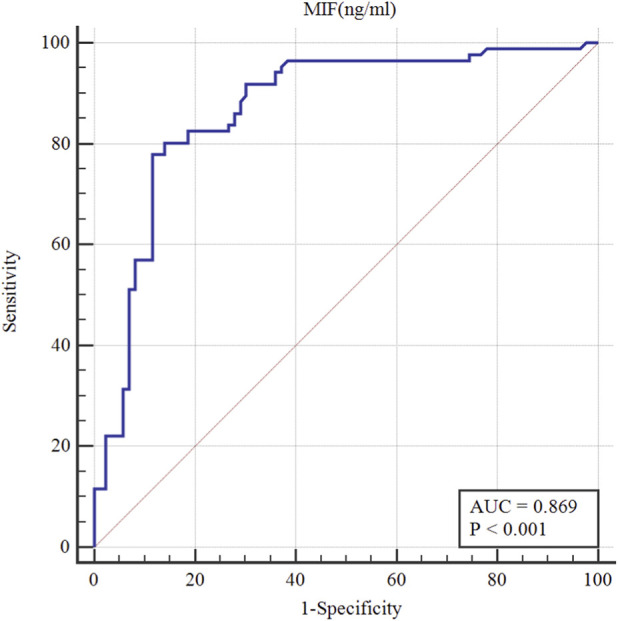
High diagnostic performance of combined plasma MIF concentrations for PMOP patients.

ROC curve analysis was conducted in the propensity score–matched population. ROC curve analysis was conducted to evaluate the diagnostic potential of plasma MIF levels for PMOP. The results revealed that the area under the curve (AUC) for MIF in the diagnosis of PMOP was 0.869 (95% CI: 0.809–0.915, p < 0.001) (Table 5; Figure 5). With the optimal cutoff value for plasma MIF concentration set at 1.282 ng/mL, We found that the sensitivity and specificity for diagnosing PMOP were 77.91% and 88.37%. This indicates that plasma MIF demonstrates a high diagnostic accuracy in distinguishing PMOP from normal bone mass status.

**TABLE 5:** Diagnostic value of MIF for Postmenopausal osteoporosis (n =172) after propensity score matching.

Variables	AUC(95%CI)	Cut-off values	Sensitivity (%)	Specificity (%)	Youden index	*P*
MIF(ng/mL)	0.869 (0.809-0.915)	1.282	77.91	88.37	66.28	0.001

p-value was significant when less than 0.05.

MIF, macrophage migration inhibitory factor.

All analyses were conducted after propensity score matching.

## 4 Discussion

Postmenopausal osteoporosis (PMOP) is a commonly occurring metabolic bone disorder in middle-aged and elderly women. With the acceleration of global population aging, its prevalence continues to increase, posing a significant risk to the health and quality of life of older individuals (Gao and Zhao, 2023; Liang et al., 2025). In this research, we systematically evaluated the expression profile of plasma MIF in PMOP and further explored its potential value as a biochemical biomarker for the diagnosis of PMOP. that patients with PMOP had significantly higher levels of plasma MIF compared to women with normal bone mass, and this difference remained consistent even after propensity score matching, indicating that the observed increase in MIF levels was not influenced by factors such as age, body mass index, or years since menopause. Moreover, plasma MIF levels were significantly negatively correlated with lumbar spine and femoral neck bone mineral density, while showing positive correlations with the bone turnover markers β-CTX and PINP. Multivariate logistic regression analysis further identified MIF as an independent risk factor for PMOP. Receiver operating characteristic (ROC) curve analysis revealed that plasma MIF exhibited favorable diagnostic performance in discriminating PMOP from normal bone mass status, highlighting its potential clinical utility as an adjunctive diagnostic biomarker for postmenopausal osteoporosis.

Inflammatory responses are increasingly acknowledged as crucial regulators of the metabolic balance in bones. MIF, a highly conserved, multifunctional proinflammatory cytokine, serves as an enhancer and prolonged activator in inflammatory processes and has been linked to the development and advancement of various inflammation-related orthopedic conditions, such as rheumatoid arthritis and ankylosing spondylitis (Bae and Lee, 2018; Onuora, 2017). Unlike molecules that directly participate in bone formation or bone resorption, MIF more accurately reflects abnormalities within the inflammation-associated microenvironment and thereby exerts regulatory effects under pathological conditions of bone metabolic imbalance. Prior research has indicated that MIF is an aglycosylated protein weighing around 12.5 kDa, and possesses a structurally conserved form, factors which contribute to its stable levels in circulation (Sobierajski et al., 2013). These properties theoretically confer advantages for MIF as a circulating biomarker, capable of reflecting disruptions in bone metabolism associated with chronic inflammation.

In regards to the regulatory pathways of bone metabolism, previous foundational research has shown that MIF has the ability to attach to the CXCR4 receptor, consequently facilitating the movement of osteoclast precursors towards sites of inflammation and intensifying their maturation and bone-resorbing function, ultimately worsening osteolytic degradation (Movila et al., 2016). Animal studies has also demonstrated that plasma levels of MIF are markedly elevated in ovariectomized (OVX) mouse models, whereas MIF-deficient mice do not exhibit significant bone loss following OVX surgery (Oshima et al., 2006). Moreover, targeted inhibition of MIF has been reported to attenuate bone loss to a certain extent (He et al., 2023). In addition, genetic polymorphisms associated with elevated MIF expression, such as the MIF-CATT7/rs755622 variants, have been shown to correlate with lower BMD and heightened fracture susceptibility among postmenopausal women (Swanberg et al., 2010). The results of the current study, which demonstrate the inverse relationship between plasma MIF levels and BMD, as well as the positive relationship with BTMs, are in line with experimental and genetic findings (Kim et al., 2016). These findings provide additional evidence for the potential role of MIF in the pathogenesis and advancement of PMOP.

Regarding BTMs, the present study demonstrated that both β-CTX and PINP were markedly increased in patients with PMOP and were identified as independent risk factors in multivariate analysis, which is consistent with previous reports. β-CTX primarily reflects the level of bone resorption, whereas PINP represents bone formation activity, and the concomitant elevation of both markers suggests a high bone turnover state in patients with PMOP (Eastell and Szulc, 2017). However, BTMs essentially reflect the dynamic process of bone remodeling, and their measurements are susceptible to various factors, including inter-individual variability, timing of sample collection, physiological conditions, and hepatic or renal function (Eastell and Szulc, 2017; Hlaing and Compston, 2014; Vasikaran et al., 2011). The relatively high biological variability of BTMs limits their clinical utility as standalone diagnostic indicators. In clinical practice, BTMs are generally interpreted in conjunction with structural assessments such as bone mineral density (LeBoff et al., 2022). In contrast, the elevated plasma MIF levels observed in this study may more directly reflect the pathological background of inflammation-associated disturbances in bone metabolism, thereby providing complementary information beyond that offered by traditional BTMs. These findings support the potential role of MIF as an adjunctive biomarker for PMOP, offering additional diagnostic insight distinct from conventional BTMs.

Notably, Previous research on the correlation between MIF and PMOP has mainly centered on descriptive association analyses, with limited systematic evaluation of its diagnostic performance. Building upon prior research, the present study employed propensity score matching and multivariate regression analysis to further strengthen the independence of the association between MIF and PMOP. This study is, to the best of our knowledge, the initial attempt to thoroughly evaluate the discriminatory potential of plasma MIF in diagnosing PMOP. ROC curve analysis demonstrated that plasma MIF achieved an AUC of 0.869, indicating a high level of diagnostic accuracy. At an optimal cutoff value, plasma MIF provided a favorable balance between sensitivity and specificity, suggesting that it may serve as a valuable adjunct to bone mineral density assessment. This is particularly relevant for populations in which DXA is not widely accessible or is unsuitable for repeated measurements. These findings offer new insights into early screening and risk stratification for PMOP.

From a clinical and translational perspective, plasma MIF could function not only as a potential indicator for PMOP but also as a hopeful focus for future interventions. Previous studies have demonstrated that MIF inhibitors can suppress osteoclastogenesis and ameliorate bone metabolic imbalance by blocking signaling pathways such as NF-κB (Jin et al., 2021; Zheng et al., 2019). Given the significant connections found in this study between plasma MIF levels, bone mineral density, and BTMs, it is proposed that targeted interventions focusing on MIF may be theoretically viable for preventing and treating postmenopausal osteoporosis. Nevertheless, the specific clinical applicability of MIF targeting has yet to be comprehensively elucidated, necessitating additional validation through well-structured animal experiments and prospective clinical research.

We must recognize several limitations of the current study. First, the cross-sectional design prevented the establishment of a temporal relationship and causal inference between elevated plasma MIF levels and the development of PMOP. Second, as a single-center study with a relatively limited sample size and number of events, the stability of the multivariate analyses may have been affected. Third, although no significant correlations were observed between plasma MIF levels and PTH or 25-OH-D3 in the present study, the underlying mechanisms were not further explored. Given that these biomarkers may influence bone metabolism through distinct biological pathways, their circulating levels may not necessarily be correlated in a cross-sectional analysis. Fourth, variability in plasma MIF measurements across different studies may exist, potentially attributable to differences in sample processing procedures, ELISA kits, and assay parameters (Bae and Lee, 2018). Future research utilizing multi-center, prospective methodologies with increased sample sizes and standardized detection protocols is necessary to further confirm the diagnostic value of plasma MIF in PMOP.

## 5 Conclusion

Using a cross-sectional analysis, this study systematically assessed the expression profile of plasma MIF and its clinical significance in PMOP among postmenopausal women. The findings indicated that patients with PMOP had significantly higher levels of plasma MIF, which were closely linked to decreased bone mineral density and abnormal BTMs. Further investigations identified MIF as an independent factor associated with PMOP and highlighted its promising diagnostic performance in distinguishing between PMOP and normal bone mass status. These findings suggest that plasma MIF, as an inflammation-related cytokine, may reflect the pathological microenvironment of abnormal bone metabolism in postmenopausal osteoporosis and holds potential value for risk assessment and adjunctive diagnosis of PMOP. This study provides novel clinical evidence supporting the involvement of MIF in PMOP, although its application as a circulating biomarker requires further validation through multicenter, prospective investigations.

## Data Availability

The raw data supporting the conclusions of this article will be made available by the authors, without undue reservation.
